# Prognostic value of transglutaminase 2 in non-small cell lung cancer patients

**DOI:** 10.18632/oncotarget.17374

**Published:** 2017-04-24

**Authors:** Zhu Chihong, Ling Yutian, Wan Danying, Jiang Ruibin, Sheng Huaying, Gu Linhui, Feng Jianguo

**Affiliations:** ^1^ Cancer Research Institute, Zhejiang Cancer Hospital, Hangzhou, Zhejiang 310022, China; ^2^ Key Laboratory Diagnosis and Treatment Technology on Thoracic Oncology, Hangzhou, Zhejiang 310022, China

**Keywords:** TG2, NSCLC, prognosis

## Abstract

Transglutaminase 2 (TG2) plays important roles in cell survival and cancer progression. In this study, we examined TG2 expression in specimen of 194 patients diagnosed with non-small cell lung cancer (NSCLC), and found that the TG2 gene expression was significantly higher in lung cancer tissues as compared to paired incisal marginal tissues or normal tissues. Our data revealed that patients with lower level of TG2 expression detected in cancer tissues had longer disease free survival and overall survival as compared to the patients with higher TG2 expression. We also found that TG2 expression level correlated to NSCLC recurrence. These results suggest a potential prognosis impact of TG2 for NSCLC patients.

## INTRODUCTION

Lung cancer remains one of the leading causes of cancer mortality worldwide [1]. The majority of lung cancer is NSCLS (non-small-cell lung cancer), and one third of these patients are diagnosed with stage III disease when curative treatment is extremely limited [2]. Despite the tremendous efforts and progress in lung cancer research, and the use of aggressive multimodal chemo- and radiotherapy, the overall treatment outcomes for these NSCLC patients remain poor, and corresponding 5-year survival rates were 54% for patients with localized tumor, 26.55% for patients with regional metastasis, and 4% for patients with distant metastasis [3].

Cancer prognostic biomarkers are patient or tumor characteristics that predict clinical outcome independent of the treatment [4], and the goal of identifying prognostic biomarkers is to define patient subpopulations with significantly different anticipated outcomes, who might benefit from different therapies. In lung cancer, clinically relevant prognostic information is provided by staging according to the database of The International Association for the study of Lung Cancer (IASLC)[5]. However, increasing evidences have suggested significant roles for more than 100 biomarkers, which may well provide some biological insight, in evaluation of patient prognosis with NSCLC [6–11]. However, most of these potential biomarkers do not reach clinical implementation, and it takes time to prove the clinical relevance for these recently identified potentially biomarkers [6].

In this study, we investigated the expressions of Transglutaminase 2 (TG2), a member of Transglutaminases family, in NSCLC tumors. Our results showed that the TG2 gene expression was significantly higher in lung cancer tissues as compared to paired incisal marginal tissues or normal tissues. The data also showed that patients with lower level of TG2 expression detected in cancer tissues had longer disease free survival and overall survival as compared to the patients with higher TG2 expression, and TG2 expression level correlated to NSCLC recurrence. Our results present here thus revealed a potential prognosis value of TG2 for NSCLC patients.

## RESULTS

### Patients’ characteristics

Among 194 patients, there were 155 men and 39 women with age ranging from 37 to 76 years old (mean 60.08±8.56). 81 cases were diagnosed with adenocarcinoma, 110 cases with squamous cell carcinoma and other 3 cases large cell carcinoma, epidermoid carcinoma and mucoepidermoid carcinoma for each. 53 cases were diagnosed at stage I, 82 cases at stage II and 52 cases at stage III. 167 cases were with family cancer history, and 168 cases were with smoking history and 167 cases with alcohol use history.

### TG2 mRNA and protein expressions in NSCLC

**Figure 1:**
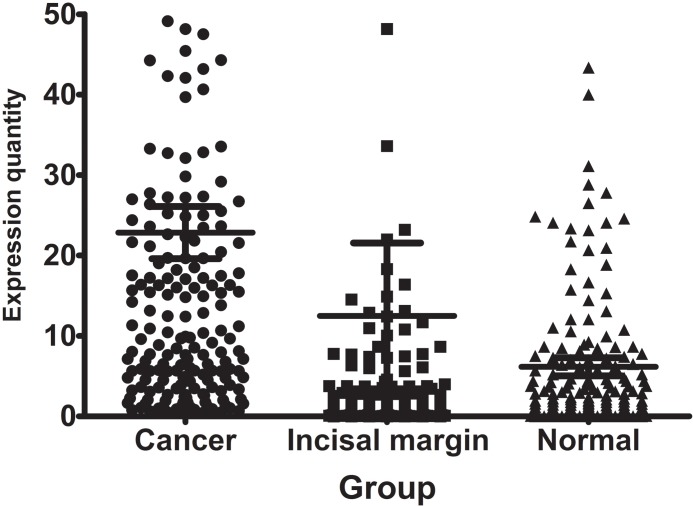
The expression of TG2 gene in paried NSCLC tumor, incisal marginal and normal tissues The difference in TG2 mRNA expression between lung cancer tissues and marginal tissues, lung cancer tissues and normal tissues, incisal marginal tissues and normal tissues were all significantly different (P all < 0.001).

The expression of TG2 gene in tumor tissues, incisal marginal tissues and normal tissues from these 194 patients were measured using real-time PCR. The relative mRNA expressions, as compared to their own actin, were 22.85±45.15, 12.49±126.33 and 6.16±15.60, respectively (Figure 1). These results showed a trend of TG2 mRNA expression towards to lower expression in lung cancer tissues, marginal tissues and normal tissues. The difference of TG2 mRNA expression in lung cancer tissues versus in marginal tissues, or in lung cancer tissues versus in normal tissues or in incisal marginal tissues versus in normal tissues were all with statistical significance (P all <0.001).

**Figure 2:**
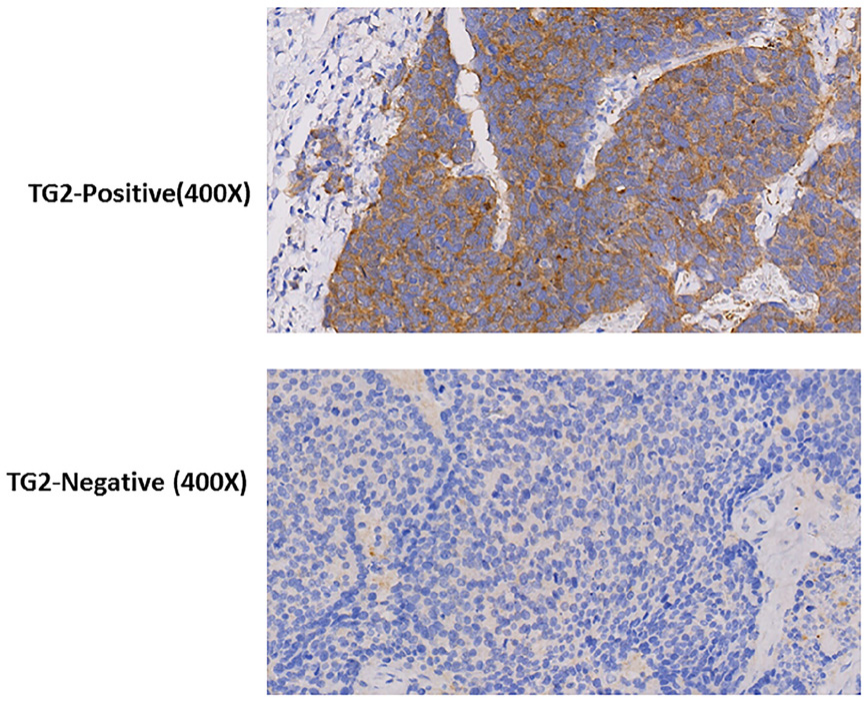
Representative images showing the immunostaining of TG2 protein in TMA

In addition, we detected positive TG2 immunostaining in 75 cases of tumor tissues (38.7%), with the IHC performed in the tissue microarray containing 194 paired specimen (Figure 2).

### Relationship of TG2 mRNA and protein expressions with Clinical-Pathological Factors in NSCLC

Nonparametric and chi-square tests showed that TG2 mRNA and protein expression were associated with NSCLC histologic type, stage and Tumor differentiation. TG2 mRNA and protein level were also significantly lower in Squamous cell carcinoma, early stage and well-differentiated (high to middle grade) tumors when comparing to adenocarcinoma, advanced stage and middle to low grade of tumors (P all <0.05), respectively (Table 1 and 2). We did not observe any correlations between expression of TG2 and clinical features of age, gender and family cancer history. Of note, histories for smoking and drinking may also correlate to TG2 mRNA level and/or protein expression.

**Table 1:** Association of TG2 mRNA level with clinicapathological factors in patients with NSCLC

Factors	Patients, n(%)	TG2	P	TG2		P
		Median (Mean, 5^th^-95^th^)		Low expressionn(%)	High expressionn(%)	
Sex			0.947			0.858
Male	155 (80.7)	11.54 (22.97,15.51-30.44)		77 (42.1)	78 (38.6)	
Female	39(19.3)	10.92 (21.61,13.74-29.47)		20(7.9)	19 (11.4)	
Age(years)			0.450			0.565
≤60	90(69.3)	4.51(4.67,4.06-5.27)		47 (34.3)	43(35.0)	
>60	104(30.7)	26.38(40.57,29.23-51.91)		50 (15.7)	54 (15.0)	
Family History			0.827			0.186
no	136(80)	10.68(20.82,16.76-24.89)		70 (43.6)	66 (36.4)	
yes	31 (16.4)	9.85(36.88(1.51-72.24)		17(6.4)	14 (10)	
Smoking			0.960			0.510
Never	39 (22.1)	9.92(21.89,13.69-30.10)		22 (9.3)	17(12.9)	
Ever/Current	129 (74.3)	11.11(24.45,15.63-33.28)		65(40.7)	64(33.6)	
Alcohol			0.212			0.329
Never	77 (44.3)	13.15(22.42,17.10-27.74)		36 (20.7)	41(23.6)	
Ever/Current	90(54.3)	9.64(25.29,13.06-37.53)		50 (27.9)	40(26.4)	
Histologic type			0.402			0.011
Squamous cell carcinoma	110 (57.1)	10.93(23.35,12.69-34.02)		46(33.6)	64(23.6)	
Adenocarcinoma	81 (35.7)	14.81(22.70,17.43-28.00)		49(12.1)	32(23.6)	
Others^a^	3 (7.1)	3.58(8.34,-16.19-32.87)		2(4.3)	1(2.9)	
Grade			0.004			***0.010***
high-middle	93 (46.4)	9.66(19.58,14.70-24.46)		60(19.3)	33(2.71)	
middle-low	88 (46.4)	18.87(35.51,12.57-38.45)		40(28.6)	48(17.9)	
Clinical stage			***0.03***			***0.018***
I	53 (37.9)	3.04(7.58,0.32-21.76)		35(22.9)	18(15.0)	
II	82(27.9)	8.02(8.60,0.14-23.08)		42 (15.7)	40(12.1)	
III	52(34.3)	12.42(14.71,0.27-24.97)		20(19.3)	32(15.0)	

*p <* .05 were highlighted in bold.

**Table 2:** Association of TG2 protein level with clinicapathological factors in patients with NSCLC

Factors	Patients, n(%)	TG2		*P*
		Negativen(%)	Positiven(%)	
Sex				0.734
Male	155 (80.7)	96 (42.1)	59(38.6)	
Female	39(19.3)	23(7.9)	16 (11.4)	
Age(years)				0.951
≤60	90(69.3)	55 (34.3)	35(35.0)	
>60	104(30.7)	64 (15.7)	40 (15.0)	
Family History				0.227
no	136(80)	76 (43.6)	60(36.4)	
yes	31 (16.4)	21(6.4)	10 (10)	
Smoking				0.007
Never	39 (22.1)	30 (9.3)	9(12.9)	
Ever/Current	129 (74.3)	68(40.7)	61(33.6)	
Alcohol				0.0001
Never	77 (44.3)	55 (20.7)	22(23.6)	
Ever/Current	90(54.3)	39(27.9)	51(26.4)	
Histologic type				0.001
Squamous cell carcinoma	110 (57.1)	56(33.6)	54(23.6)	
Adenocarcinoma	81 (35.7)	61(12.1)	20(23.6)	
Others^a^	3 (7.1)	2(4.3)	1(2.9)	
Grade				***0.015***
high-middle	93 (46.4)	63(19.3)	30(2.71)	
middle-low	88 (46.4)	44(28.6)	44(17.9)	
Clinical stage				***0.001***
I	53 (37.9)	42(22.9)	11(15.0)	
II	82(27.9)	48 (15.7)	34(12.1)	
III	52(34.3)	22(19.3)	30(15.0)	

### Relationship of TG2 mRNA and protein expressions with disease progression and overall survival in NSCLC

**Figure 3:**
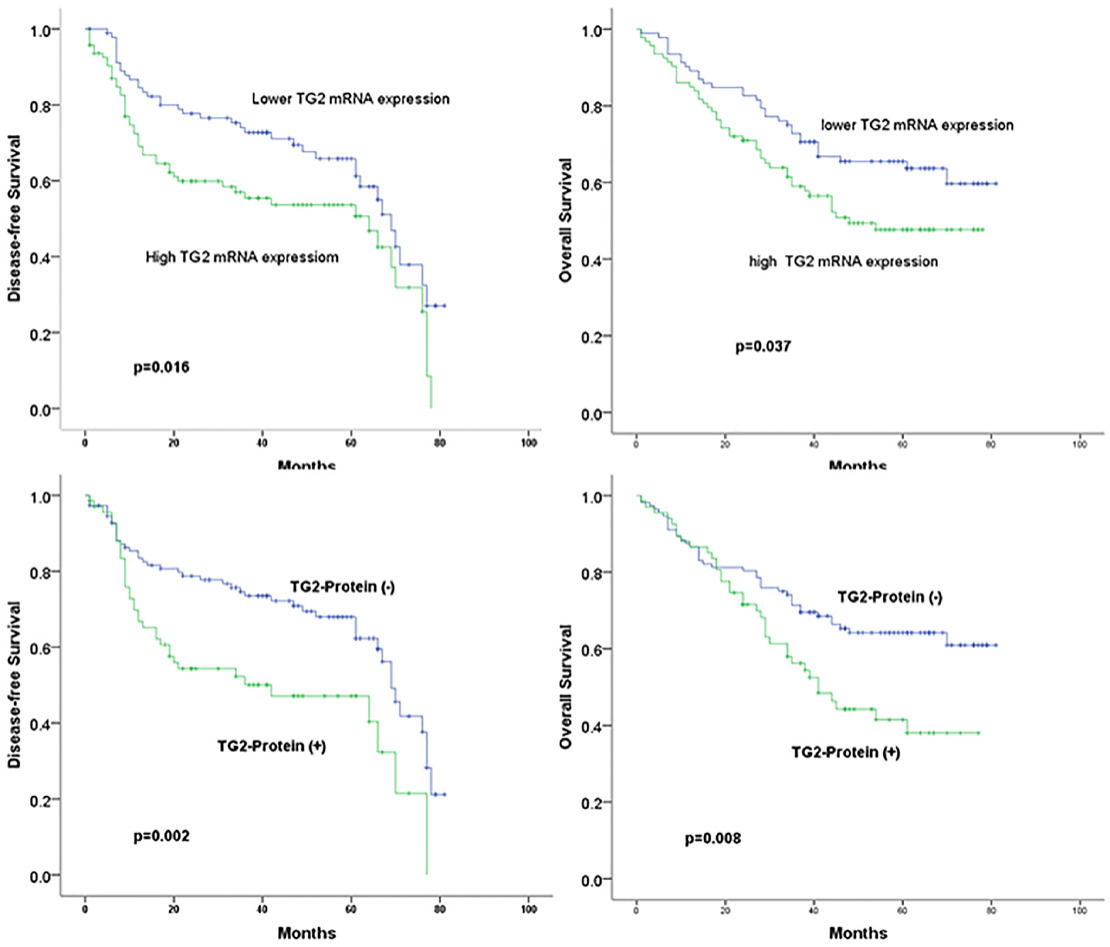
Correlation of TG2 expression with PFS and OS in NSCLC Top: correlations of TG2 mRNA expression with PFS and OS in NSCLC; bottom: correlations of TG2 protein expression with PFS and OS in NSCLC.

With Kaplan–Meier analysis and log-rank test, our data showed a strong correlation between the TG2 mRNA and/or protein expressions and the disease progress (tumor relapse and metastasis). The lower level of relative expression of TG2 mRNA and protein indicated dramatically longer disease-free survival (DFS) as compared to the patients with tumors expressing higher levels of TG2 mRNA and protein expression (P<0.05). Patients with tumors expressing lower levels of TG2 mRNA and protein also had better overall survival (OS) rates when comparing to the patients with tumors expressing higher levels of TG2 mRNA and protein (P<0.05). (Figure 3).

### Survival analysis

By using Cox regression analysis, we found that TG2 mRNA and protein expression significantly correlated with the DFS and OS. Our data also indicated that Gender, clinical stages and smoking history of these NSCLC patients may also correlated with the DFS and OS. However, no evidence showed that clinical features of age, drinking history, family cancer history, histologic type and tumor grade had correlations with the DFS and OS (Table 3–4).

**Table 3:** Survival model by Cox regression analysis for DFS and OS of TG2-mRNA and clinicopathological characteristics

Variable	DFS			OS		
	Exp(B)	95%CI	P Value	Exp(B)	95%CI	P Value
TG2-mRNA	2.02	1.23-3.35	***0.006***	2.07	1.20+3.58	***0.009***
Age	1.16	0.71-1.91	0.540	1.02	0.58-1.77	0.954
Sex	0.10	0.03-0.32	***0.000***	0.14	0.04-0.55	***0.005***
Smoking	0.69	0.34-1.40	***0.001***	0.34	0.12-0.95	***0.039***
Drinking	1.18	0.69-2.00	0.551	1.37	0.77-2.45	0.290
FamilyHistoy	0.85	0.34-1.39	0.290	1.68	0.87-3.27	0.125
Histologic type	1.45	0.87-2.36	0.140	0.85	0.48-1.50	0.572
Grade	0.85	0.52-1.40	0.529	0.72	0.42-1.25	0.245
Clinical stage	1.74	1.05-2.91	***0.033***	4.01	2.23-7.2	***0.000***

**Table 4:** Survival model by Cox regression analysis for DFS and OS of TG2-protein and clinicopathological characteristics

Variable	DFS			OS		
	Exp(B)	95%CI	P Value	Exp(B)	95%CI	P Value
TG2-Protien	2.29	1.39-3.79	***0.001***	2.60	1.50-4.49	***0.001***
Age	1.24	0.76-2.04	0.384	1.12	0.65-1.93	0.679
Sex	0.09	0.03-0.31	***0.000***	0.11	0.03-0.43	***0.002***
Smoking	0.20	0.08-0.51	***0.001***	0.26	0.09-0.74	***0.011***
Drinking	1.67	0.69-1.99	0.243	1.34	0.75-239	0.325
FamilyHistoy	0.66	0.32-1.33	0.243	1.46	0.75-2.84	0.267
Histologic type	1.34	0.82-2.19	0.242	0.79	0.45-1.38	0.404
Grade	0.91	0.59-1.48	0.70	0.77	0.45-1.32	0.347
Clinical stage	1.71	1.03-2.84	***0.040***	3.72	2.09-6.62	***0.000***

## DISCUSSION

Transglutaminase 2 (TG2) is a ubiquitous multifunctional mammalian protein that catalyzes the formation of intermolecular isopeptide bonds between glutamine and lysine residues of several proteins [14, 15]. The enzymatic activity of TG2 is allosterically regulated by several factors, including guanine nucleotides, Ca+2, and redox potential [16]. TG2 has been found to be involved in a diverse range of biological processes, including apoptosis, membrane signaling, cell adhesion and extracellular matrix formation, and elevated expression of TG2 was detected in various forms of cancer. For example, studies have reported that cancer cells failing to apoptosis expressed high levels of TG2 [17], and increased expression of TG2 could prolong cell survival by preventing apoptosis [18]. Studies further demonstrated that TG2 could interact with the proteins such as pRb, integrins and fibronectin to induce activation of cell survival and antiapoptotic signaling pathways and prevent cancer cells from apoptosis [19, 20]. Other studies reported that cancer cells with developed resistance to chemotherapeutic drugs exhibited extraordinary high levels of TG2 expression compared with the parental cell line from which they were derived [21–24], and downregulation of TG2 expression or inhibition of TG2 enzymatic activity can convert chemoresistance in cancer cells [25, 26]. In our previous study, we also reported that treatment with TG2 inhibitor increase radiosensitivity in human lung cancer cells [27]. In addition, TG2 expression was also reported to induce Epithelial-to-Mesenchymal Transition (EMT) which result in the increased invasiveness of cancer cells [28, 29]. These evidences thus suggest that TG2 may play important roles in cancer cell survival and cancer metastasis or recurrence during clinical treatment. Of note, Jeong JH et al. has recently reported that TG2 expression correlated to the DFS and progression-free survival (PFS) for NSCLC in a small cohort containing enrolling 29 patients [30]. In another study with a larger cohort containing 429 NSCLC cases, Choi CM., et al. has also reported significant correlation between strong TG 2 expression and shorter DFS in the non-adenocarcinoma subtype of NSCLC, but they did not identify similar correlation in the adenocarcinoma subtype [31]. Our data form a cohort with 194 NSCLC cases, however, showed strong correlations of TG2 expression with DFS and with OS in overall NSCLC patients diagnosed with both non-adenocarcinoma subtype and adenocarcinoma subtype of cancers. Nevertheless, these observation and reports suggest a potential clinical impact of TG2 as a prognostic biomarker for NSCLC patients. However, further studies, including the studies for correlation of TG2 expression with other prognostic marker for NSCLC, are needed.

## MATERIALS AND METHODS

### Primary tumor tissues and patients

194 primary tumor tissues and paired adjacent nontumor tissues or incisal marginal tissues were collected from surgical specimens of NSCLC patients between March 2006 and April 2010 in Zhejiang Cancer Hospital. All the patients did not receive any anticancer therapy such as chemotherapy and radiotherapy, or other treatments prior to surgery. Paired nontumor samples were obtained at least 2 cm distant from the tumor and incisal marginal tissues were obtained within 2 cm distant from the tumor. Informed consent was obtained from all patients, and the study was reviewed and approved by the Clinical Research Ethics Committee of the Zhejiang Cancer Hospital. The histological diagnosis was based on the classification criteria for lung tumors of the World Health Organization and International Association for the Study of Lung Cancer (WHO/IASLC). The tumor stage was defined according to the seventh edition of tumor-nodemetastasis (TNM) classification.

### Follow-up

All patients received standardized follow-up with patients themselves or family members up to March 15, 2015. Among all 194 patients, 82 patients had relapse during the follow-up, with 75 patients died, 5 patients had no record of distal metastasis and 15 patients had no record of overall survival.

### RNA extraction

Lung tissues (about 2 g) were first homogenized using Qiagen Tissue Lyser (Qiagen, Valencia, CA, USA) and then total RNA were extracted using DNA/RNA/Protein mini kit according to the manufacturer's instructions (Qiagen). The concentration and purity of RNA were measured with nanodrop 1000 (NanoDrop, Wilmington, DE, USA).

### Real-time reverse transcription-PCR

A reverse transcription reaction was performed using 1 μg of total RNA with Prime Script RT Reagent (200) Kit (Takara No. DRR037A). The RNA expression levels of TG2 were determined by real-time RT-PCR using SYBR® Premix Ex Taq™ (Tli RNaseH Plus)(Takara No. DRR420A) and ABI 7500 Real-time PCR System (Life Technology, Foster City, CA, USA). The primers used in this study were designed based on the sequences information from NCBI database, and synthesized by Takara Bio Inc. The forward primer for TG2 was 5′- CCTGATCGTTGGGCTGAAG-3′ and the reverse primer was 5′- TCGGCCAGTTTGTTCAGGTG-3′. The length of PCR product was 144 bp. The β-actin was used as internal control of RNA integrity, with the forward primer of 5′- TGGCACCCAGCACAATGAA-3′ and the reverse primer of 5′- CTAAGTCATAGTCCGCCTAGAAGCA-3′. The length of PCR product for β-actin was 186 bp. RT-PCR was carried out using the following conditions: 10 min at 95°C, followed by 40 cycles at 95°C for 15 s and 60°C for 1 min. A melting curve analysis program was used to verify the authenticity of the PCR products by their specific melting temperatures (Tm) after amplification according to manufacturer's instructions. The cycle threshold (Ct) values were determined by the SDS software that was installed in the real-time PCR machine. The relative TG2 gene expression in different lung tissues was calculated using 2^−ΔΔCt^ method as compared to its own actin level which was described before [12]. The median of the RNA expression was calculated and used as a threshold to distinguish the higher and lower expression within the factor groups.

### Tissue microarray (TMA) and Immunohistochemistry (IHC)

TMA blocks were established as described previously [13]. Briefly, TMA blocks were obtained with 1.0 mm diameter of representative regions, identified with hematoxyline-eosin (H.E) staining, of each case from 194 patient surgical specimen. The cores were carefully selected on H.E stained sections and inserted into new paraffin blocks using Tissue Arrayer Minicore (ALPHELYS, Plaisir, France). The sections with 5 μm thickness were then deparaffinized with xylene, and washed by 100% ethanol, 90% ethanol, 70% ethanol and finally distilled water. The sections were primed for antigen retrieval in citrate buffer (pH 6.0) using microwave heating for 5 minutes cycle. Sections were incubated with 1:80 diluted Anti-TG2 antibody (EP2957, Abcam, Cambridge, MA, USA) over night at 4°C, followed by incubation with biotin labeled goat anti-mouse IgG and HRP-conjugated streptavidin (DAKO, USA) for immunostaining. The slides were scored according to staining intensity, -(negative), + (weak positive), ++ (positive), +++ (strong positive);Positive cell rates were scored from 0 to 3, 0(<5%), 1(5%-25%), 2(25%-50%), 3(>50%). The final grades were scored according to the formula, (+) %x1 + (++) %x2+ (+++) %x3, with +(<1.0), ++(1.0-1.5), +++(>1.5).

### Statistical analysis

The relative expression of TG2 was described as mean and standard deviation and compared with paired t test and Chi-square test. Survival curves were obtained by using the Kaplan–Meier method. Disease-free survival (DFS) was defined from the date of definitive surgery to the date of local or distant progression, death or the date of last follow-up. Overall survival (OS) was calculated as the time from pulmonary surgery to death or censoring. The survival curves were compared by using the log-rank test. All the statistical calculations were performed by using SPSS 13 (Chicago, IL, USA). p <0.05 was considered statistically significant.

## Abbreviations

TG2: Transglutaminase 2
NSCLC: non-small cell lung cancer
